# A survey on the early identification and brief intervention for hazardous and harmful alcohol consumption in primary health care: the European Alcohol Measures for Public Health Research Alliance (AMPHORA) project

**DOI:** 10.1186/1940-0640-8-S1-A66

**Published:** 2013-09-04

**Authors:** Emanuele Scafato, Claudia Gandin, Silvia Ghirini, Lucia Galluzzo, Sonia Martire, Lucilla Di Pasquale, Alfredo Cuffari

**Affiliations:** 1 National Observatory on Alcohol, National Center for Epidemiology, Surveillance and Health Promotion - CNESPS, National Institute of Health - ISS, Rome, Italy; 2 National Society of Medical Education for the General Practitioners – SNAMID, Italy

## 

In Europe, hazardous and harmful alcohol consumption is the second highest risk factor for premature death and disability and of many medical diseases and conditions. In daily professional practice, Primary health care (PHC) professionals frequently deal with patients who consume alcohol at risk for their health. In 2011 in Italy, 23.9% of men and 6.9% of women aged 11+ years have been estimated as hazardous drinkers—more than 8 million individuals. In the context of the European Alcohol Measures for Public Health Research Alliance (AMPHORA) project, the National Observatory on Alcohol of the National Centre for Surveillance, Prevention and Health Promotion (NOA-CNESPS) of the Italian National Institute of Health (ISS), in collaboration with the *“Società nazionale di aggiornamento per il medico di medicina generale”* (SNAMID), a scientific society for the training of general practitioners (GPs), carried out a survey on national knowledge, attitudes, and perceptions of GPs on the use of EIBI for hazardous and harmful alcohol consumption. The aim of the survey, performed in 6 EU countries, was the identification of the main barriers and facilitators for the implementation of EIBI in PHC. Most of participants did not receive training on alcohol and alcohol-related problems: 24% did not receive training at all, 26% "less than 4 hours", 28% "from 4 to 10 hours", and only 7% “more than 40 hours” across the professional life. Only 31.9% (the lowest percentage among participating European countries) said they were familiar with standardized screening instruments, of which only half used them in their clinical practice. Furthermore, only 37.5% were familiar with brief intervention techniques. In Italy, EIBI strategy for hazardous and harmful alcohol consumption is included in nearby all strategic public health documents. Nevertheless, EIBI is still offered sporadically. Discussion on barriers, facilitators and on perceptions of GPs on the use of EIBI are presented.

